# Facile generation of *Hermes* insertion mutants in prototrophic *Candida glabrata* for use in nutrient-limited environments

**DOI:** 10.1128/spectrum.00848-26

**Published:** 2026-05-29

**Authors:** Anna M. Zaeske, Abigail A. Harrington, Timothy J. Nickels, Andrew N. Gale, Winston Timp, Nicole Alayo, Yasmine Hassoun, David S. Perlin, Erika Shor, Kyle W. Cunningham

**Affiliations:** 1 Department of Biology, Johns Hopkins University 228291, Baltimore, Maryland, USA; 2 Department of Biomedical Engineering, Johns Hopkins University 171259, Baltimore, Maryland, USA; 3 Center for Discovery and Innovation, Hackensack Meridian Health 721734 , Nutley, New Jersey, USA; 4 Department of Medical Sciences, Hackensack Meridian School of Medicine 576909, Nutley, New Jersey, USA; 5 Georgetown University Lombardi Comprehensive Cancer Center 66634, Washington, DC, USA; CNRS-Inserm-Université Côte d'Azur, Nice, France

**Keywords:** Tn-seq, *Candida glabrata*, intestinal colonization

## Abstract

Transposon mutagenesis coupled with deep sequencing (Tn-seq) is currently being deployed in microbial eukaryotes, including the opportunistic yeast pathogen *Candida glabrata*, for functional genomics research. This method depends on the generation of highly diverse pools of transposon insertion mutants to cover all genes while minimizing the presence of markers and remnants of engineering. Up to now, pools of *Hermes* transposon insertion mutants in *C. glabrata* were generated in uracil-requiring *ura3∆* auxotrophs, limiting their use in nutrient-restricted environments, such as those of the host. Indeed, we found that *ura3∆* mutants were outcompeted by *URA3+* prototrophs during colonization of the mouse gastrointestinal tract. To avoid using auxotrophs in Tn-seq experiments, a new scheme was developed for generating prototrophic pools of *Hermes* insertion mutants. The scheme involved introducing a recessive cycloheximide resistance mutation in the chromosomal *RPL28* gene, which did not alter fitness during mouse colonization. When implemented in several different strains of *C. glabrata*, high insertion densities were obtained, and differences in subtelomeric chromatin compaction were observed that correlated with natural variation in the silencing gene, *SIR3*. However, all the strains lacked insertions in the *PDR1* and *CDR1* genes, which are necessary for resistance to cycloheximide and other antifungals. We directly tested the effect of *pdr1∆* mutants and found that they exhibited moderate fitness defects in the gastrointestinal tract of mice even in the absence of antifungals. Thus, the new scheme easily generates high-quality pools of insertion mutants in prototrophic *C. glabrata* with only minor and knowable limitations.

**IMPORTANCE:**

Treatment of fungal infections may be improved by a deeper understanding of the genetic mechanisms of colonization within host organisms. Current approaches to deep genetic sequencing in eukaryotic microbes often involve engineered components that have significant biases, minimize microbial complexity, or alter the normal *in vivo* fitness of opportunistic fungal pathogens. This study designs a new method for developing transposon insertion mutants in *Candida glabrata* that does not innately introduce altered fitness in mouse models of gastrointestinal tract infection. This scheme is also portable across strains and possibly even fungal species. The findings show that the new method can be used in this pathogenic yeast to yield highly complex pools and reliably identify genetic components of colonization in mouse models of infection.

## INTRODUCTION

Transposon mutagenesis and deep sequencing (Tn-seq) methods have been utilized extensively in bacteria for forward genetic screens designed to reveal genes that are essential or become essential in different environments (1, 2). These methods all take advantage of large pools of mutants, each bearing a random transposon insertion in its genome, and employ next-generation sequencing of insertion sites for *en masse* analysis. The scope and granularity of Tn-seq can reveal important genetic features in initial pools, such as essential genes that are underrepresented with transposon insertions in the starting pools. Upon experimental manipulation, transposon pools can unveil even broader implications regarding the genetic mechanisms of fitness. When the environment is altered, some disrupted genes may become significantly under- or overrepresented in the population and provide information on biological mechanisms. This can contribute key insights into the biological determinants of microbial fitness under varied conditions, including within a host environment (3).

Tn-seq methods are just starting to be utilized in eukaryotic microbes. The model yeasts *Saccharomyces cerevisiae* and *Schizosaccharomyces pombe* have been extensively mutagenized with the housefly *Hermes* transposon (4–7). More recently, the *mini-Ac/Ds* transposon from maize has been exploited in *S. cerevisiae* to produce extremely high complexity pools (8). A major advantage of Tn-seq methods is the portability to non-model organisms with limited genetic tools. Haploid derivatives of the normally diploid human pathogen, *Candida albicans*, have been mutagenized with both the *piggyBac* transposon from moth (9) and *mini-Ac/Ds* (10). The pathogen *Cryptococcus neoformans*, which is naturally haploid, has been mutagenized with *mini-Ac/Ds* (11). In those species, little or no gain-of-expression effects were observed or reported, unlike in *S. cerevisiae*, where intrinsic enhancer activity can produce gain-of-expression when *mini-Ac/Ds* is integrated in 5′ non-coding sequences (8). *piggyBac* mutagenesis of the extremely AT-rich genome of *Plasmodium falciparum*, the major malarial parasite in humans, is currently being developed for Tn-seq applications (12, 13). *piggyBac* was also launched successfully in the naturally haploid yeasts *Candida auris* (14) and *Candida glabrata* (15). These *Candida* species are distantly related to *C. albicans* and are increasing rapidly in clinical incidence due to innate and easily acquired resistance to most classes of antifungals. Additionally, both the *piggyBac* and *Hermes* transposons have been launched successfully in the industrial yeast *Yarrowia lipolytica* (16, 17). Finally, *Hermes* mutagenesis of *C. glabrata* was also achieved (18, 19), resulting in pools of insertion mutants with much higher complexity and greater gene coverage than those achieved using *piggyBac* (15).

Of the abovementioned transposons, *Hermes* exhibits a moderate preference for insertion at TxxxxA sites (4), whereas *mini-Ac/Ds* lacks such sequence preferences, resulting in a higher overall complexity of the pools of insertion mutants (18). The *piggyBac* transposon strongly prefers TTAA sequences at insertion sites and therefore produces pools with lower complexity and lower gene coverage than the others (20). Additional types of insertion bias unique to eukaryotic cells can further diminish the overall complexity of the pools, such as occlusion of the DNA by nucleosomes, transcription factors, centrosomes, and telomeres, as well as compaction of the DNA by heterochromatin formation. With proper analytical tools, such effects can be exploited to gain new insights into gene function, gene domains, chromosome organization, and more.

*C. glabrata* is an opportunistic pathogenic yeast that usually acts as a commensal in the human gastrointestinal (GI) tract but can also cause infections, or candidiasis, that may advance to life-threatening systemic infections like candidemia in immunocompromised individuals. To explore the genetic mechanisms behind its advance to pathogenesis, *C. glabrata* has been analyzed by Tn-seq using *piggyBac* and *Hermes* transposons, with each method exhibiting unique benefits and drawbacks. The *piggyBac* mutagenesis of *C. glabrata* involved four sequential genome modifications: (i) introduction of *his3∆* and (ii) *trp1∆* mutations, as well as integration of (iii) an inducible transposase expression cassette (*TRP1+*) and (iv) the donor *piggyBac* transposon (*HIS3+*) at ectopic sites (15). The resulting pools of insertion mutants were prototrophic but suffered from relatively low complexity and incomplete gene coverage. Additionally, the parent strain for *piggyBac* mutagenesis carried a tandem duplication of 60 genes (131 kbp) on chromosome K that is not present in any natural isolates of *C. glabrata* (21). Meanwhile, the *Hermes* mutagenesis scheme involved the introduction of *ura3∆* in the genome, followed by transformation with a centromeric plasmid bearing an inducible transposase expression cassette, the selectable *NAT1* marker in the transposon, and the counter-selectable *URA3* gene (18). The *Hermes* insertion mutants were then enriched by selection with nourseothricin plus 5-fluoroorotic acid (FOA), which selects against cells that retain the unexcised or excised plasmids. Though the *Hermes* pools exhibited much higher complexity and gene coverage, the *ura3∆* mutation was present in all of the derived strains. The uracil auxotrophy could potentially impact the results of transposon studies under conditions where uracil availability may be limiting, such as within the host.

Here, we show that *ura3∆* mutants of *C. glabrata* do indeed exhibit a mild fitness defect in a widely utilized mouse model of GI tract colonization. To avoid this concern, we developed a novel scheme for enriching *Hermes* insertion mutants in *URA3+* (prototrophic) strains of *C. glabrata*. The scheme utilized a modified plasmid launchpad and cycloheximide (CHX), rather than FOA, to deplete the plasmid from the pool of insertion mutants. The resulting pools exhibited high complexity, though the *PDR1* and *CDR1* genes were no longer covered with insertions as these genes are known to be essential for CHX resistance (19). We measured the fitness of *pdr1∆* mutants during colonization of the mouse gastrointestinal tract, where they showed moderate fitness defects as detected by declining representation in fecal pellets over time after co-gavage with wild-type parent strains. This improved scheme for generating pools of transposon insertion mutants is easily transferable to different strains of *C. glabrata* and is expected to generate results in a wider range of conditions. We used this scheme to generate prototrophic pools of insertion mutants in three *C. glabrata* strains from different clades, CBS138, BG2, and DPL1021, and found that subtelomeric DNA was highly accessible to transposon insertions in CBS138 and DPL1021 but not BG2 (21). Thus, this work develops a new scheme of Tn-seq with the *Hermes* transposon and a prototrophic strain of *C. glabrata* that generates complex pools, is portable across strains, and has the power to demonstrate key genetic elements both *in vitro* and *in vivo* with minimal and known biases.

## MATERIALS AND METHODS

### *C. glabrata* strains and plasmids

All strains of *C. glabrata* utilized in this study are listed in Table S1. The *rpl28-Q38E* mutation was introduced into wild-type strains by transformation with a 1.1 kb PCR product obtained by amplification of the *rpl28-Q38E* locus of strain AGY25 (19) using primers listed in Table S2. Colonies that grew on yeast peptone dextrose (YPD) medium containing 5 µg/mL CHX were picked, purified, and authenticated by PCR and DNA sequencing using appropriate primers (Table S2). To generate a transposon launchpad plasmid that restores sensitivity to CHX, the original pCU-MET3-Hermes plasmid was digested with StuI plus ApaI to remove a segment of the *URA3* gene and ligated to a similarly digested PCR product obtained by amplification of the wild-type *RPL28* gene locus of BG2 using appropriate primers (Table S2). The resulting pCR-MET3-Hermes plasmid and the parent pCU-MET3-Hermes plasmid were transformed into *C. glabrata* strains using the lithium acetate method and selected on YPD agar medium containing 100 µg/mL nourseothricin.

### Mouse gavage and fitness testing

Individual *C. glabrata* strains were revived from frozen stocks on YPD agar medium, and single colonies were regrown in YPD medium. Revived pools and strains were pelleted, washed once in sterile PBS, and resuspended to 10^9^ viable cells per mL in sterile PBS. The suspensions of mutants were mixed 1:1 with suspensions of wild-type parent strains. Oral gavage was performed using 100 µL of each suspension (approximately 10^8^ cells) on 6-week-old female CF-1 mice (Charles River Laboratory) that had been treated subcutaneously every day from day −2 to day 6 with 320 mg piperacillin-tazobactam (PTZ)/kg of body weight (AuroMedics Pharma LLC, East Windsor, NJ, USA) to minimize native intestinal bacterial microbiota. On days 1, 4, and 7 post-gavage, several fecal pellets were collected from each mouse and homogenized in sterile PBS. For competition experiments, the homogenates were serially diluted in PBS and plated onto YPD + PTZ plates to quantify total CFU per sample (burden), and plates with countable numbers of colonies were replica plated to YPD + hygromycin (HYG) or YPD + CHX to quantify mutant CFU per sample. CFU counts from five replicate mice were averaged, charted, and fit by nonlinear regression to a simple exponential decay equation (KaleidaGraph v5).

### Broth microdilution assays

*C. glabrata* strains transformed with plasmids were grown to saturation in YPD medium containing 100 µg/mL nourseothricin and then diluted 2,000-fold into the same medium containing varying concentrations of CHX in 96-well dishes. After 24-h incubation at 30°C, optical density was measured at 600 nm using a microplate reader (Smartreader 96T, Accuris Instruments). Raw OD_600_ data were fit by nonlinear regression to a standard sigmoid equation in which the dose of CHX causing a 50% inhibition of growth (IC_50_) was estimated.

### Generation and enrichment of pools of insertion mutants

Pools of *Hermes* insertion mutants were generated in *C. glabrata* strains bearing pCR-MET3-Hermes using the exact same protocol as before with the original pCU version of the plasmid launchpad (18). Briefly, a single colony was suspended in 100 mL SCD + NAT medium lacking methionine and cysteine to derepress the expression of transposase, then divided into 40 aliquots of 2.5 mL each to minimize jackpot events, and cultured at 30°C with vigorous shaking. After 3 days, individual cultures were pooled, pelleted, resuspended in 600 mL fresh YPD + NAT medium, and cultured for 24 h at 30°C to permit loss of excised plasmids. The cells were then pelleted, resuspended in an equal volume of YPD + NAT + CHX medium, and cultured for 1 day as before to begin enrichment of cells bearing transposon insertions and lacking the plasmid launchpad. In the next two rounds of enrichment, 60 mL of the previous cultures was harvested, pelleted, resuspended in 600 mL YPD + NAT + CHX, and cultured for 24 h each. Periodically, 1 mL samples were removed, serially diluted, and plated on YPD, YPD + NAT, and YPD + NAT + CHX plates to determine levels of enrichment by relative CFUs. Final pools were pelleted, resuspended in 60 mL of 1× YEP plus 15% glycerol, and stored at −80°C in 4 mL aliquots until use.

### Tn-seq methods

Aliquots of CHX-enriched pools of insertion mutants in strains BG2r and CBS138r were thawed, pelleted, and revived by regrowth in 10 mL YPD medium. Cells were pelleted, washed in sterile water, and then genomic DNA was extracted, purified, sheared by sonication, ligated to splinkerette adapters, PCR amplified in two stages, and sequenced (MiSeq, Illumina Inc.) exactly as described previously (19). A similar method was developed for sequencing of insertion mutants in pools from strain DPL1021r on a different instrument (Aviti, Element Biosciences Inc.). The main changes were (i) ligation of splinkerette adapters that lack indexes, (ii) attachment of indexes and capture sequences in the PCR step, and (iii) utilization of custom sequencing primers (Table S2). Detailed protocols for Aviti sequencing are available on request. The MiSeq and Aviti output files were handled identically. High-quality reads were demultiplexed, trimmed, mapped to the BG2, CBS138, and DPL1021 reference genomes (22–24), filtered to remove multimapping and hybrid reads, and then tabulated site-wise and gene-wise as before (18). Complexity (midLC) of each sequenced sample was estimated using a back-sampling approach (18), which estimates the number of Tn-seq reads that map to half as many unique sites in the reference genome used. Pearson correlation coefficients were calculated from normalized gene-wise counts (Table S3)

## RESULTS

### Fitness in the mouse GI tract declines with the *ura3∆* mutation, but not the *rpl28-Q38E* mutation or drug resistance cassettes

**Fig 1:**
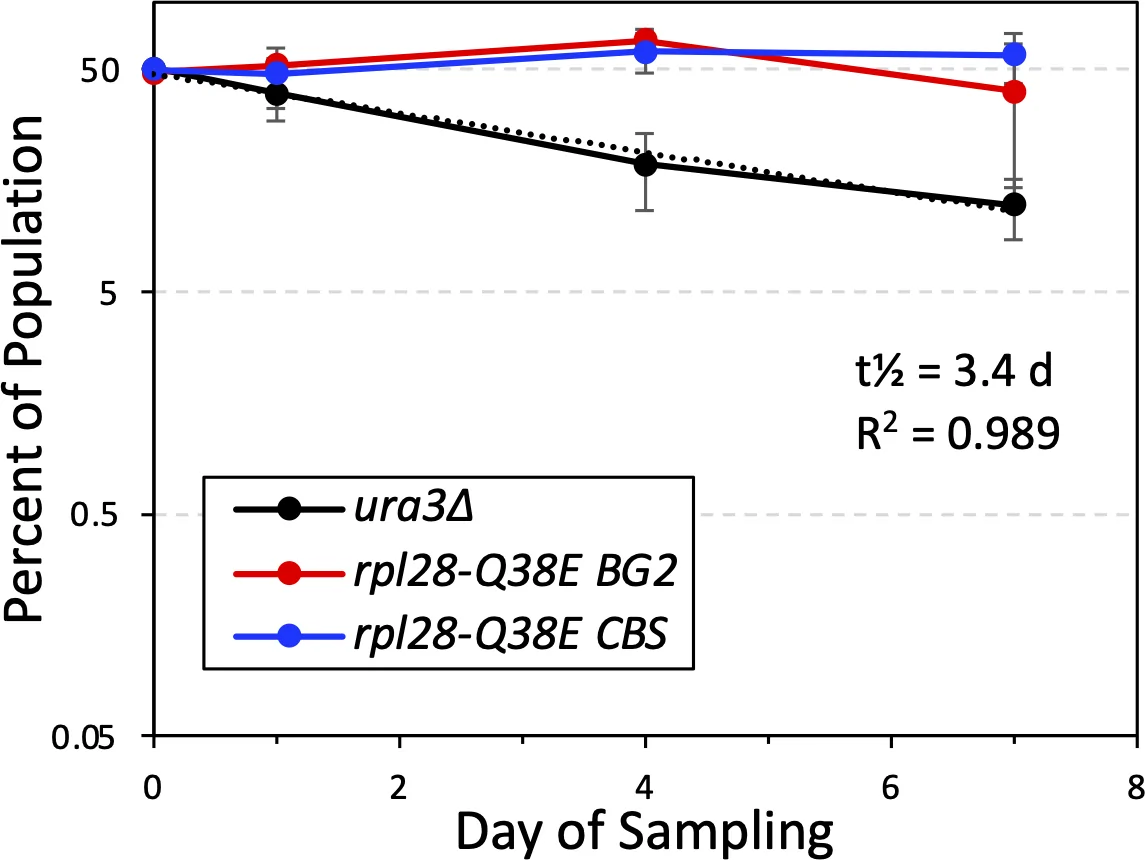
Competitive fitness of *C. glabrata* mutants in the mouse gastrointestinal tract. Cultures of wild-type strain BG2 were mixed 1:1 with cultures of BG2u (*ura3∆*; black symbols) or BG2r (*rpl28-Q38E*; red symbols) and orally gavaged into five mice that had been treated with PTZ antibiotics. Fecal pellets were collected on days 1, 4, and 7 post-gavage, and fungal load was measured, followed by replica plating to determine the percentage of mutant colonies relative to the total. The data from all five mice were averaged (±SD). Competitive half-life was determined for BG2u by nonlinear regression using an exponential decay equation. A similar experiment was performed using wild-type strains CBS138 and CBS138r (*rpl28-Q38E*; blue symbols).

The *ura3∆* mutation remains in the background of all pools of *Hermes* insertion mutants in *C. glabrata* generated to date (19, 21). To determine whether the *ura3∆* mutation confers a fitness disadvantage in the mouse GI tract, the wild-type strain BG2 (prototrophic) was mixed 1:1 with isogenic BG2u (*ura3∆* mutant, auxotrophic). The mixture was gavaged orally into five mice that had been pretreated with piperacillin-tazobactam antibiotic to limit competition between endogenous microbiota and *C. glabrata* (25). Fresh fecal pellets were obtained from each mouse periodically over the course of a week, homogenized, diluted, plated on YPD + PTZ media, and incubated. After *C. glabrata* colonies appeared, the percentage of *ura3∆* colonies was determined by replica plating. The frequency of *ura3∆* mutant colonies steadily declined relative to the prototrophic parent strain, described by an exponential decay equation with a competitive half-life of 3.4 days (Fig. 1, black symbols). These findings suggest that the *ura3∆* auxotrophic mutants initially colonized the mouse GI tract with wild-type efficiency but, due to a mild fitness deficit in this environment, were then overtaken by wild-type cells.

Therefore, a different scheme was devised for the enrichment of mutant cells bearing transposon insertions that utilized CHX instead of FOA in the enrichment step. In this strategy, a CHX-resistant *rpl28-Q38E* mutation (19) was introduced into the genome of the prototrophic *C. glabrata* strain, while the plasmid used for launching the *Hermes* transposon was modified to contain the dominant CHX-sensitive *RPL28* gene in place of the *URA3* gene (Fig. S1). To determine whether the genomic *rpl28-Q38E* mutation impacts fitness in the mouse GI tract, parent strains BG2 and CBS138 were analyzed in competition with their respective CHX-resistant derivatives. In both strain backgrounds, the *rpl28-Q38E* mutants remained at ~50% frequency of the total population over the entire 7-day period (Fig. 1, red and blue symbols). Therefore, this CHX-resistant mutation did not impact relative fitness in the mouse GI tract, providing a neutral marker that could be exploited in the generation of enriched pools of transposon insertion mutants for use in nutrient-limited environments.

### *Hermes* insertional mutagenesis in prototrophic strains of *C. glabrata*

**Fig 2:**
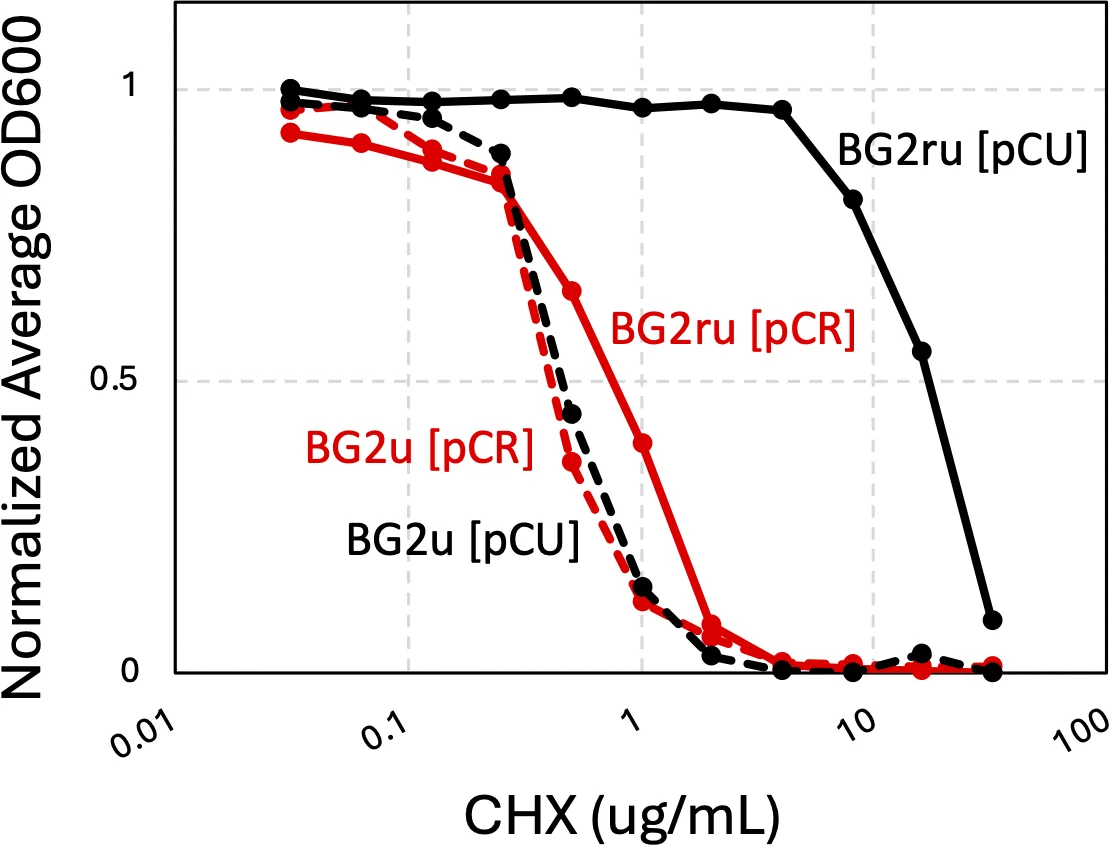
Reversion of CHX resistance by plasmids bearing *RPL28*. Strains BG2u and BG2ru (*rpl28-Q38E ura3∆*) carrying pCU-MET3-Hermes or pCR-MET3-Hermes plasmids were tested for resistance to CHX in broth microdilution assays (YPD medium containing nourseothricin). Averages of three replicates (±SD) are shown.

To achieve successful transposon enrichment with this new scheme, the plasmid launchpad bearing *RPL28* must substantially diminish the resistance of the *rpl28-Q38E* host strains to CHX. To quantify this effect, the original launchpad plasmid bearing *URA3* (pCU-MET3-Hermes) and the new launchpad plasmid bearing *RPL28* (pCR-MET3-Hermes) were each transformed into BG2u and the *rpl28-Q38E* derivative (BG2ru). Resistance to CHX was quantified in broth microdilution assays in YPD medium containing nourseothricin to maintain selection for the *NAT1* marker on the plasmids. The *RPL28* plasmid diminished CHX resistance of the *rpl28-Q38E* strain by over 20-fold relative to the *URA3* plasmid, nearly to the levels observed in the BG2u parent (Fig. 2). To test possible strain-to-strain variation of these parameters, six additional strains from different clades of *C. glabrata* were engineered to contain the CHX-resistant *rpl28-Q38E* mutation and the plasmids. Relative to the *URA3* plasmid, the *RPL28* plasmid diminished CHX resistance in all these strains by factors ranging from 5- to 21-fold (Table 1). Though the magnitude of diminished resistance and precise IC_50_s varied somewhat between the different strains, the scheme for CHX enrichment of insertion mutants seemed feasible and scalable across strains.

**TABLE 1:** Reversion of CHX resistance by plasmids introduced into different strains

Strain	Clade	CHX IC_50_ (µg/mL)		Ratio
		pCR	pCU	
BG14	7	0.8	16.5	20.4
CBS138	5	0.6	2.9	5
DPL1021	4b	0.5	3.7	6.9
P35-2	6a	0.6	4.4	6.8
EF1237Blo1	4a	0.8	5.1	6.3
M7	1	0.9	11.1	12.2
M6	7	1	20.6	21

To implement the new scheme, two independently generated *rpl28-Q38E* derivatives of BG2 were transformed with pCR-MET3-Hermes, and pools of *Hermes* insertion mutants were generated in synthetic SCD-cys-met medium to induce transposase expression (19). A four-step enrichment strategy was then implemented, where the saturated cultures were pelleted, resuspended in YPD medium containing 5 µg/mL CHX plus 100 µg/mL nourseothricin, and shaken. At each step, the percentage of double-resistant cells in the population was determined by plating assays. The level of enrichment achieved at each step was similar for the replicate pools, ultimately achieving 85% and 92% enrichment by the final step (Fig. S2). After extracting genomic DNA and then amplifying, sequencing, and mapping the insertion sites as before (19), 12% and 8% of all sequence reads from pool1 and pool2 mapped to the unexcised plasmid launchpad, indicating enrichment using CHX was less effective than using FOA (<1%). However, transposon insertions were found at over 587,000 unique sites in the genome, representing an average density of 1 insertion site every ~17 bp when essential and unmappable segments are removed. Over 187,000 of these sites (32%) were shared in the two replicate pools, and the number of reads at these shared sites was highly correlated (*R*^2^ = 0.815, Table S3). The two new CHX pools also exhibited high complexity (midLC > 172,000 and 133,000; Fig. S3), comparable to pools generated previously using the FOA method of enrichment (19, 21).

### Gene-wise comparisons between enrichment methods

**Fig 3:**
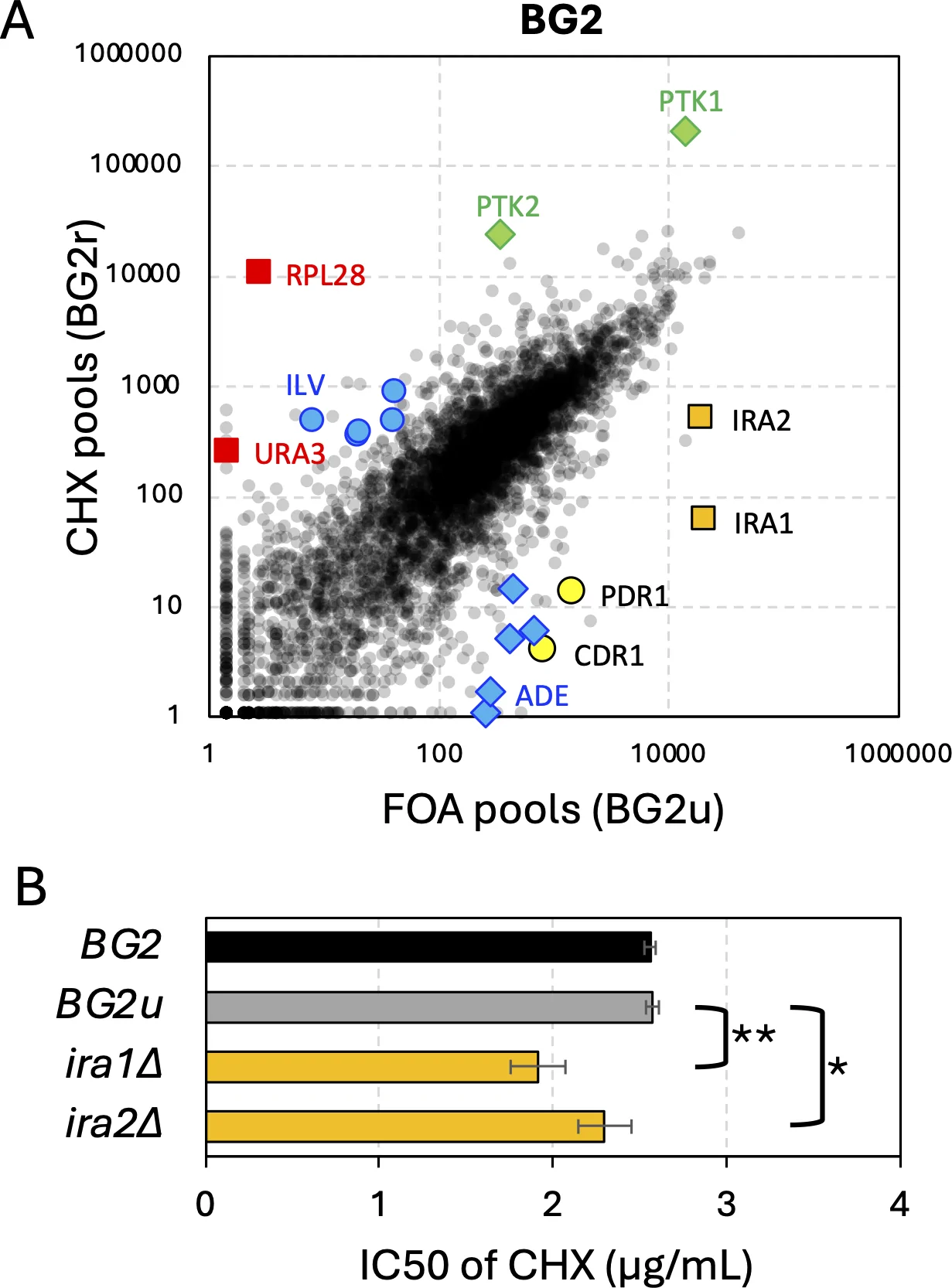
Gene-wise comparison of CHX-enriched and FOA-enriched pools of *Hermes* insertion mutants. (**A**) Pools of insertion mutants in strains BG2u and BG2r were analyzed by Tn-seq and tabulated gene-wise. All annotated genes (gray circles) are shown with specific genes (colored symbols) highlighted and labeled. (**B**) Resistance to CHX was quantified for the indicated strains using broth microdilution assays. Bars indicate the average IC_50_ obtained from four replicates (±SD). *P < 0.05; **P < 0.005 (Student’s *t*-test).

Tn-seq data from the two new CHX-enriched pools were combined, tabulated gene-wise, and compared to Tn-seq data from FOA-enriched pools. A strong correlation was observed between CHX- and FOA-enriched pools, though many outliers were also evident (Fig. 3A). As expected, the *URA3* and *RPL28* genes were outliers due to their presence on the plasmid launchpads of their respective methods (red squares). For unknown reasons, five genes involved in branched chain amino acid biosynthesis (*ILV1*, *ILV2*, *ILV5*, *ILV3*, and *BAT1;* blue circles) were overrepresented, and five genes involved in adenine biosynthesis (*ADE4, ADE5,7, ADE6, ADE8,* and *ADE16;* blue diamonds) were underrepresented by transposon insertions in the CHX-enriched pools relative to the FOA-enriched pools (Fig. 3A). Additionally, insertions in two redundant protein kinases (*PTK1* and *PTK2*; green diamonds) were highly overrepresented in the CHX pools, while insertions in two inhibitors of RAS-PKA (*IRA1* and *IRA2*; orange squares) were overrepresented in the FOA pools (Fig. 3A). To test whether *IRA1* and *IRA2* regulate resistance to CHX or FOA, *ira1∆* and *ira2∆* mutants were generated and compared to BG2 and BG2u strains in broth microdilution assays. The *ira1∆* and *ira2∆* mutants exhibited significantly lower resistance to CHX relative to the BG2 and BG2u parent strains (Fig. 3B). Finally, the *PDR1* and *CDR1* genes were strongly underrepresented with transposon insertions in the CHX pools (Fig. 3A, yellow circles) along with *GAL11-A* (Table S3). These genes were shown previously to function in a regulatory network that increased resistance to CHX as well as other antifungals (19, 26, 27). Thus, a small number of genes exhibited unequal representation with transposon insertions in the different enrichment schemes.

**Fig 4:**
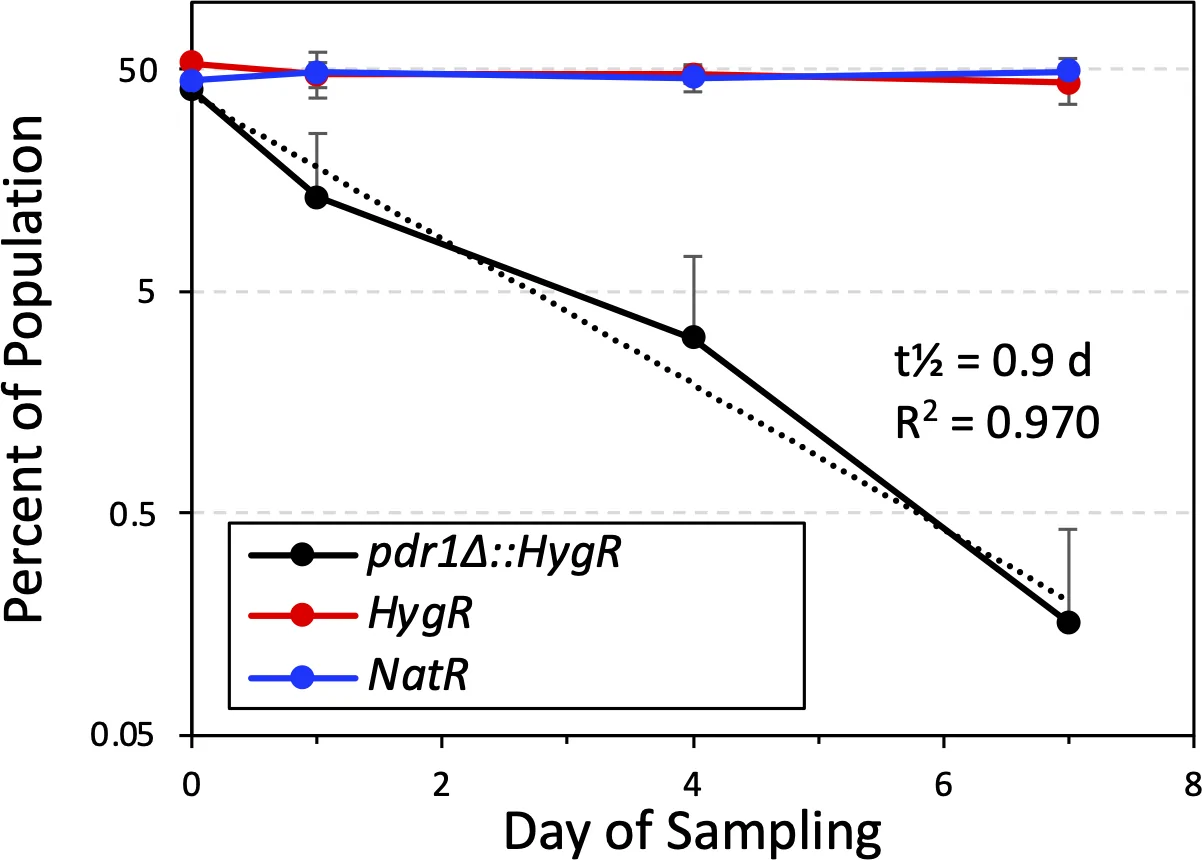
Competitive fitness of *pdr1∆*, HygR, and NatR mutant strains in the mouse gastrointestinal tract. Wild-type BG2 was mixed 1:1 with *pdr1∆::HygR* (black), *HygR* (red), or *NatR* (blue) derivatives, gavaged into five antibiotic-treated mice each, and fecal pellets were analyzed for CFU and mutant frequency by replica plating as described in Fig. 1.

The poor representation of *PDR1* in CHX-enriched pools renders it untestable in future Tn-seq screens, such as colonization of the GI tract. To test whether *PDR1* promoted colonization of the mouse GI tract, a *pdr1∆::HygR* mutant was mixed 1:1 with the BG2 parent strain, gavaged into antibiotic-treated mice, and its relative abundance in fecal pellets was determined as before. Interestingly, the *pdr1∆::HygR* mutant initially colonized the GI tract but was significantly out-competed by wild type with a competitive half-life of only 0.9 days (Fig. 4, black symbols). In contrast, the introduction of *HygR* and *NatR* expression cassettes into neutral locations of BG2 had no impact on competitive fitness in these conditions (Fig. 4). These findings unexpectedly suggest that *PDR1* function can increase the relative fitness of *C. glabrata* in the GI tract.

### Insertion mutants in prototrophic strains CBS138 and DPL1021

Pools of *Hermes* insertion mutants were also generated in *rpl28-Q38E* derivatives of the *C. glabrata* type strain CBS138 (also known as ATCC 2001) and strain DPL1021 (also known as ATCC 90030) using the same CHX enrichment scheme as above for strain BG2. Though the CBS138r pool contained 22% of Tn-seq reads mapping to the unexcised plasmid launchpad, the complexity of genome insertions was the highest observed to date (midLC > 132,000; Fig. S3). As expected, this pool contained very low numbers of insertions mapping to *PDR1* and *CDR1* and higher numbers of insertions mapping to *PTK1* and *PTK2* (Fig. S4). The DPL1021r pool exhibited lower complexity (midLC > 37,000) when mapped to the DPL1021 reference genome (Fig. S3). The *PTK1* and *PTK2* genes in DPL1021r were very strongly overrepresented with transposon insertions (Table S3), suggesting that the complexity of the DPL1021r pool may have been very high initially but declined due to excessive enrichment of mutants conferring resistance to CHX.

**Fig 5:**
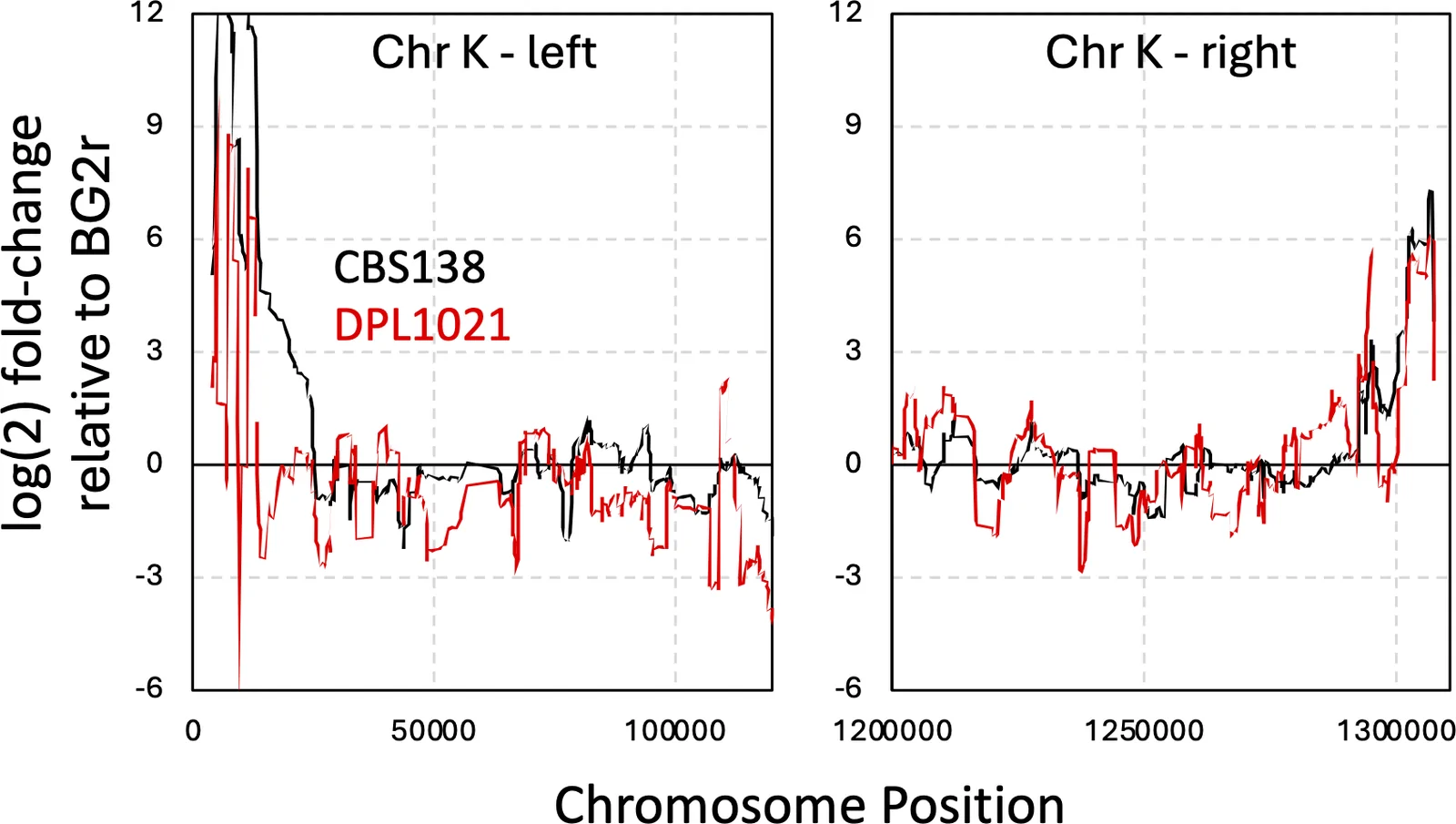
Subtelomeres of CBS138r and DPL1021r contain far more transposon insertions than BG2r. Pools of *Hermes* insertion mutants in strains BG2r, CBS138r, and DPL1021r were sequenced, mapped to the BG2 reference genome, and tabulated at each site. The log2 fold change was charted for a sliding 501-site window along each chromosome of DPL1021r (red) and CBS138r (black) relative to BG2r. Zoom-in of the left and right ends of chromosome K is depicted. Full data for all chromosomes are shown in Fig. S4.

Earlier studies have shown large differences between BG2 and CBS138 in terms of compaction of chromatin near the telomeres and silencing of subtelomeric genes (21, 28). CBS138 and BG2 belong to *C. glabrata* clades V and VII, respectively, whereas DPL1021 (ATCC 90030) belongs to clade IVb and is slightly closer to CBS138 than to BG2 (29). To determine whether DPL1021 contained CBS138-like or BG2-like chromatin in subtelomeres, a sliding window analysis was performed across each chromosome, comparing the frequency of transposon insertions in the DPL1021r and CBS138r pools relative to that of the BG2r pool. The results showed much higher densities of transposon insertions in the subtelomeres of both DPL1021r and CBS138r pools relative to the BG2r pool, suggesting that both contain open subtelomeric chromatin (Fig. S5). The effect is easily visualized after zooming in on individual subtelomeres, such as the left and right ends of Chromosome K (Fig. 5), where greater numbers of insertions are evident in both DPL1021r and CBS138r relative to BG2r. Earlier studies have shown that silencing of subtelomeric genes in BG2 depends on a specific sequence in the *SIR3* gene product, which is absent in CBS138 (30). The *SIR3* silencing sequence (N268, I503) was only found in BG2 and other members of clade VII, while DPL1021 and representatives from six other clades of *C. glabrata* contained the non-silencing sequence (H268, L503; see Fig. S6). Therefore, compaction of subtelomeric DNA and silencing of subtelomeric genes may be restricted to BG2 and closely related strains within clade VII of *C. glabrata*.

## DISCUSSION

There is a great need to understand how pathogenic eukaryotes have evolved the ability to colonize different niches, move between niches, and cause diseases. Tn-seq can provide unbiased insights into these processes by quickly revealing genes and gene networks that regulate such behaviors in the pathogens. This study develops a facile method to generate complex pools of *Hermes* transposon insertion mutants in prototrophic strains of *C. glabrata* that can be utilized for Tn-seq studies in animals and other nutrient-limited conditions where auxotrophic markers may be problematic. The method utilizes CHX instead of FOA for enrichment of cells with transposon insertions and depletion of cells that retain the plasmid launchpad. Other methods enrich by selecting directly for prototrophy that arises after excision and repair of the chromosome-based launchpad. The prototrophic pools generated by the new *Hermes* scheme were almost sixfold more complex than those produced by the *piggyBac* scheme (15), resulting in much higher coverage of the genome. However, several genes required for CHX resistance (e.g., *PDR1*, *CDR1*, and *GAL11-A*) contained few transposon insertions in the new enrichment scheme, and therefore, these genes would not be surveyed in Tn-seq experiments. Despite this limitation, the prototrophic pools can be generated easily in multiple strains of *C. glabrata* and utilized in a wide variety of applications to reveal genes that control fitness in a vast array of conditions. Other types of strain-to-strain variation can also be revealed, such as segmental duplications, large chromosomal inversions, and different states of chromatin compaction at subtelomeres.

This study also advances an approach to quantify competition, or relative fitness, of a mutant strain of *C. glabrata* relative to a wild-type control strain during colonization of the mouse GI tract. In this approach, the test and control strains are cultured separately, mixed 1:1, and gavaged into antibiotic-pretreated mice. Fecal pellets are collected at different times post-gavage, plated to determine fungal loads (31, 32), and then the deviation from 1:1 representation is quantified by replica plating the resulting colonies to appropriate selective test medium. Mutations in the genome that confer resistance to CHX and expression cassettes that confer resistance to hygromycin and nourseothricin all caused no deviation from the original 1:1 ratio, while the *ura3∆* and *pdr1∆* mutants both declined over the 7-day study interval relative to the control. The declines could be quantified by fitting an exponential decay equation to estimate competitive half-lives, which were 3.4 and 0.9 days in these specific mutants and were indicative of “mild” and “moderate” fitness deficiencies in these environments. Mutants with high fitness deficiencies would be detectable as well, particularly if sampling of fecal pellets were more frequent and if the starting ratios were varied. Moreover, mutants with fitness advantages could be quantified simply by inverting the ratio prior to curve fitting. With additional information, such as the number of generations or doubling times *in situ*, competitive half-lives could be converted to standard measures of fitness. This approach offers more sensitivity and utilizes fewer mice than the traditional approach, where test and control strains are gavaged separately into mice and fungal burden is quantified later, typically at just one time point. Additionally, this simple approach may be able to distinguish initial colonization defects (evident in the initial, but not later, time points) from fitness defects (evident on all time points).

Competition experiments showed that *C. glabrata* mutants lacking *PDR1* appeared to colonize the GI tract of antibiotic-treated mice just as well as the wild-type parent strain but were slowly outcompeted during long-term colonization (competitive half-life = 0.9 days). The mechanism by which *PDR1* promotes fitness of *C. glabrata* in the mouse GI tract is not yet known but could involve the increased expression of ABC transporters, lipid flippases, or other target genes that have been shown previously to confer resistance to antifungals and xenobiotics (33–35). The fitness defects of *pdr1∆* mutants in this environment may indicate a reduced ability to defend against toxins secreted by host cells or the residual microbiome in the GI tracts of mice. Alternatively, the *pdr1∆* mutants may have lower uptake of nutrients due to lower expression of target genes such as *AUS1*, which promotes sterol uptake and fitness of *C. glabrata* in serum and disseminated infections (36, 37). The diet of animals was recently shown to alter colonization of the GI tract by different mutants of *C. albicans* (38), so findings from just one experimental condition should be interpreted cautiously.

Though *C. albicans* can stably colonize the GI tract of mice that have not been pretreated with antibiotics (38), *C. glabrata* and many other *Candida* species colonize better when endogenous microbial communities have been knocked down with antibiotics in the GI tract of antibiotic-treated mice (25). This system offers an opportunity to study relative fitness between different strains of a species and even between different species of *Candida* in a model system with clear relevance to human health. *Candida* colonizing the human GI tract has been shown to serve as a major endogenous source for subsequent invasion of the bloodstream and dissemination to vital organs, causing life-threatening infections (39). The availability of Tn-seq in *C. glabrata* could shed light on gene networks required for its colonization, proliferation, competition with other strains and species, and host interactions in a highly tractable mammalian model.

## Data Availability

The authors affirm that all data necessary for confirming the conclusions of the article are present within the article, figures, tables, and repository. Raw sequencing reads used in this study were deposited at the NCBI Sequence Read Archive (SRA) with the BioProject ID: PRJNA1460454. Additional metadata are available upon request.

## SUPPLEMENTAL MATERIAL

Supplemental figures spectrum.00848-26-s0001.pdf Fig. S1 to S6.

Supplemental tables spectrum.00848-26-s0002.xlsx Tables S1 and S2.

Table S3 spectrum.00848-26-s0003.xlsx Tn-seq comparisons across schemes and strains.

ASM does not own the copyrights to Supplemental Material that may be linked to, or accessed through, an article. The authors have granted ASM a non-exclusive, world-wide license to publish the Supplemental Material files. Please contact the corresponding author directly for reuse.
