# Supplementary material for: Facile generation of Hermes insertion mutants in prototrophic Candida glabrata for use in nutrient-limited environments
Source: Microbiol Spectr. 2026 May 29;14(7):e00848-26. doi: 10.1128/spectrum.00848-26 (PMC13340276; doi:10.1128/spectrum.00848-26)
Supplement: Supplemental figures — Fig. S1 to S6. [file spectrum.00848-26-s0001.pdf]

Figure S1 – Zaeske et al

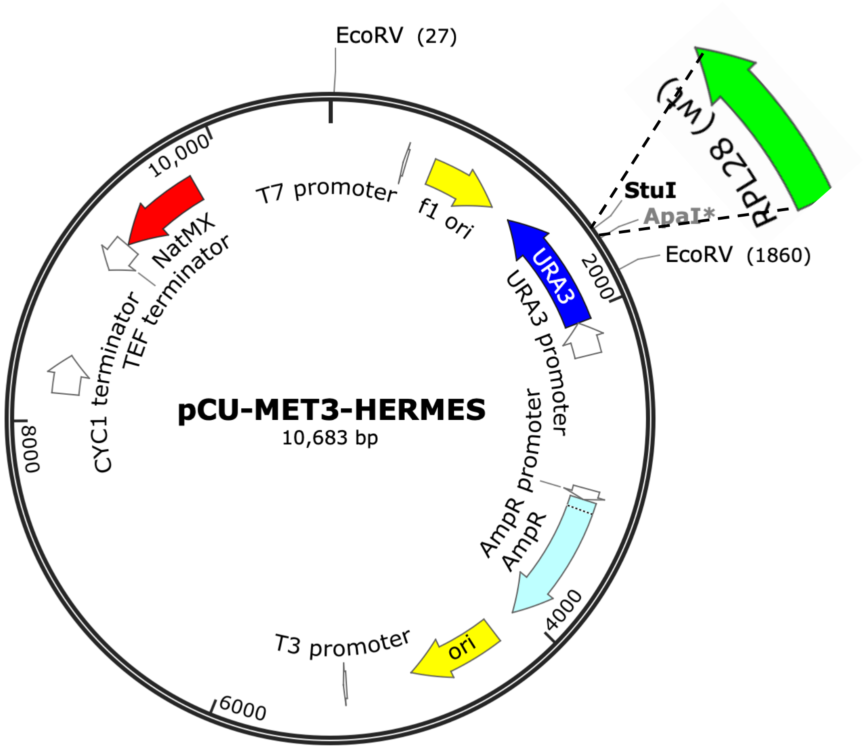

Figure S1. Construction of pCR-MET3-Hermes from pCU-MET3-Hermes.

Figure S2 – Zaeske et al

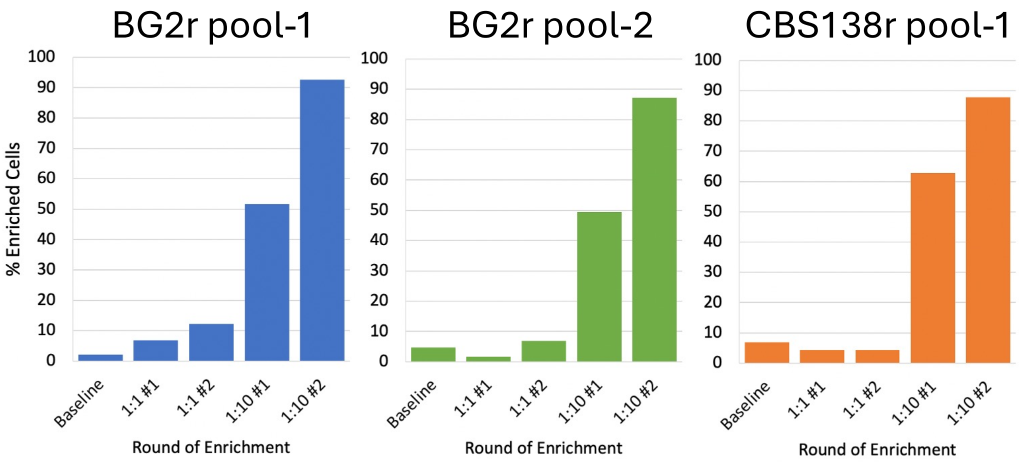

Figure S2. Efficiency of enrichment using CHX. Transposition was induced in strains BG2r and CBS138r bearing pCR-MET-Hermes and the resulting cultures were subjected to four rounds of enrichment in medium containing CHX and nourseothricin. After each round, aliquots were removed, diluted, and plated for single colonies on medium containing nourseothricin and then replica plated to medium containing nourseothricin plus CHX. The percentage of CHX-resistant colonies is shown.

Figure S3 – Zaeske et al

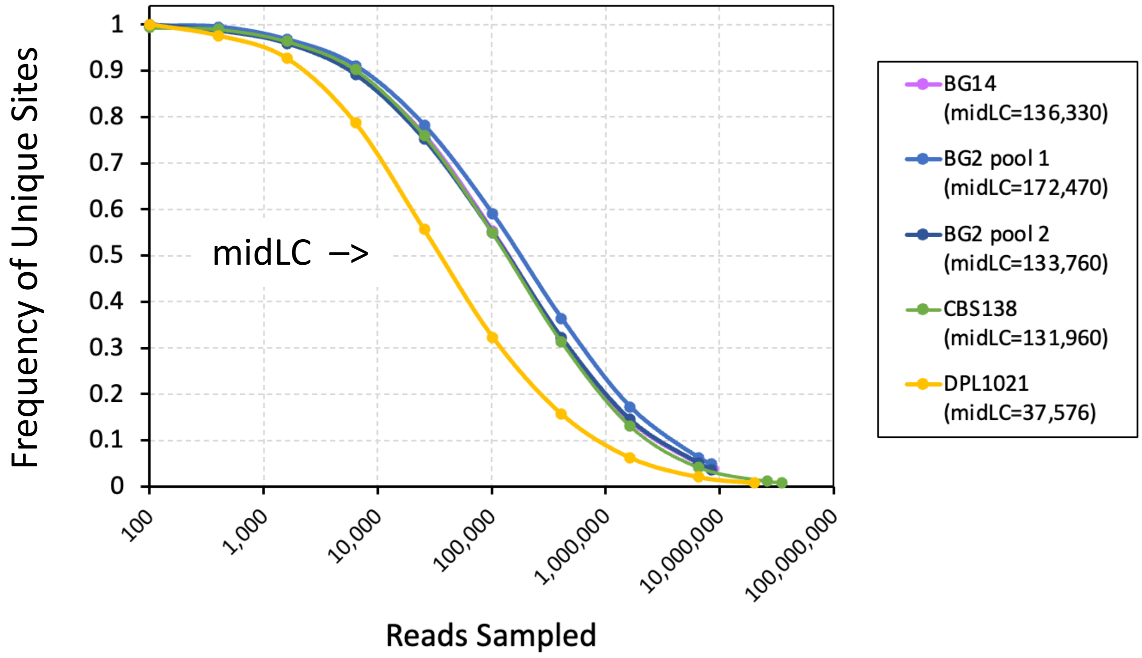

Figure S3. Estimation of mid-library complexity (midLC) of CHX-enriched pools in strains BG2r (pool-1, blue), BG2r (pool-2, dark blue), CBS138r (green), and DPL1021 (yellow) and of FOA-enriched BG2u (pool-3) was determined by back-sampling the mapped reads from each pool to different depths and charting the frequency of unique sites at each depth (average of 3 samplings). Data were fit to 2-parameter sigmoid equations to estimate the number of reads sampled when 50 percent of the sites are unique (Gale et al [2020]).

Figure S4 – Zaeske et al

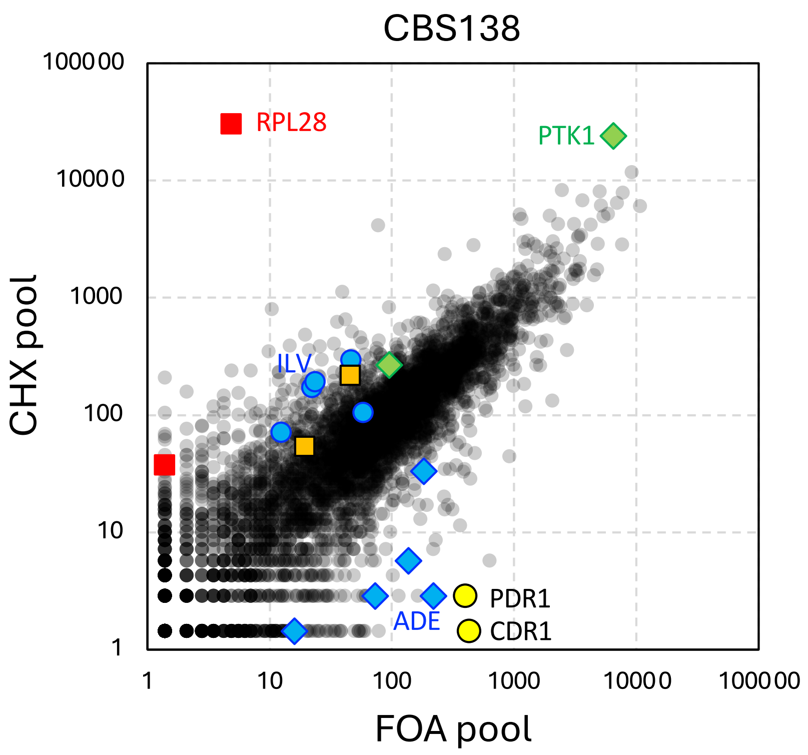

Figure S4. Gene-wise comparison of CHX-enriched and FOA-enriched pools of Hermes insertion mutants in strain CBS138. Pools of insertion mutants in strain CBS138u (x-axis) and CBS138R (y-axis) were analyzed by Tn-seq and tabulated gene-wise. All annotated genes (gray circles) are show with specific genes (colored symbols) highlighted and labeled.

Figure S5 – Zaeske et al

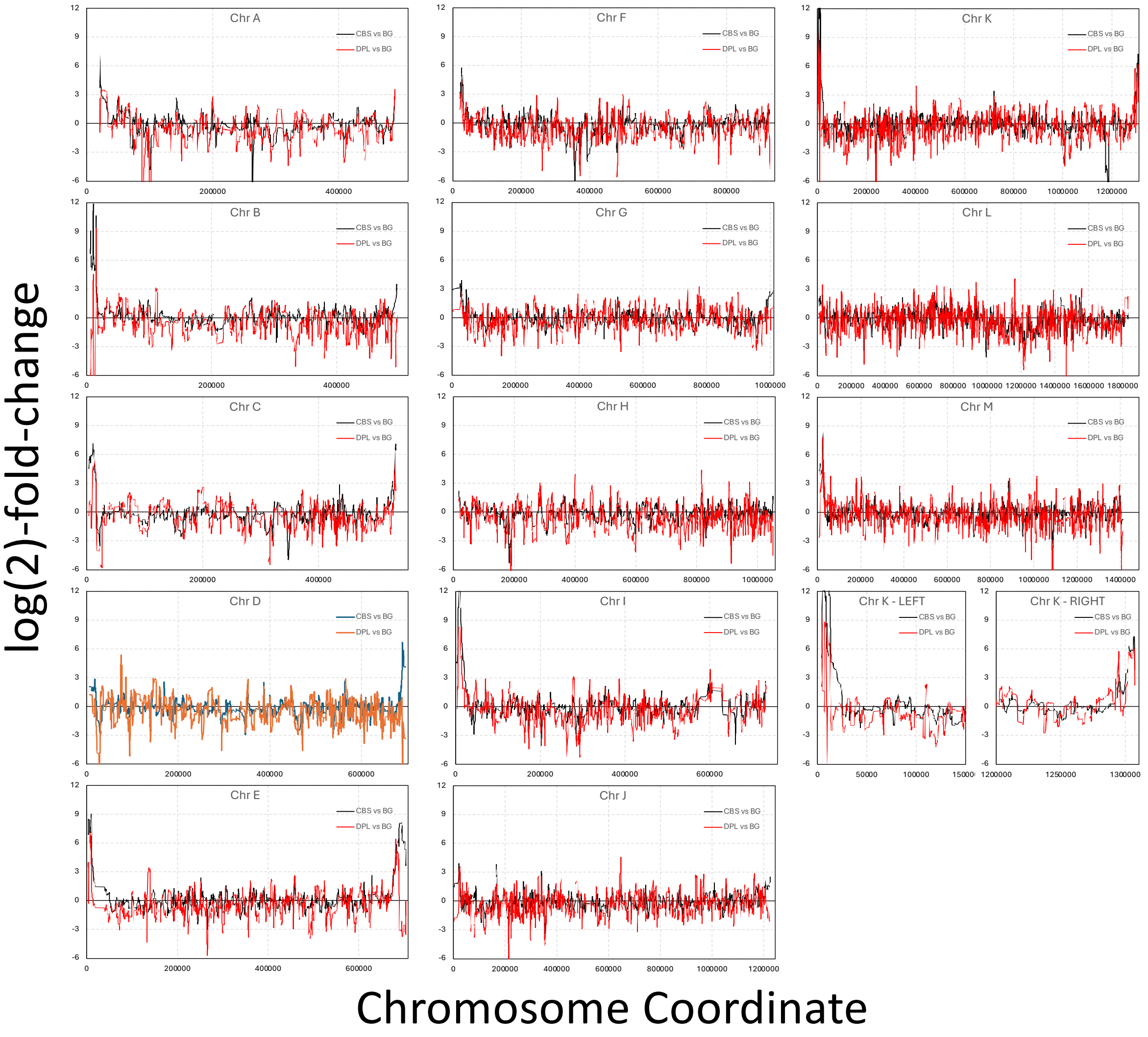

Figure S5. Sliding window analysis of *Hermes* transposon insertions in strains CBS138r (black) and DPL1021r (red) relative to BG2r for all 13 chromosomes as described in Fig. 5

Figure S6 – Zaeske et al

SIR3 codon#

Clade	Strain	13	236	268	309	371	386	397	421	426	486	503	534	536	597	679	739	769	872	919	980	998
5	CBS138	**I**	**T**	**H**	**T**	**T**	**I**	**G**	**L**	**T**	**E**	**L**	**D**	**E**	**T**	**D**	**I**	**N**	**P**	**T**	**I**	**D**
5	CAS08-0027	.	.	.	.	.	.	.	.	.	.	.	.	.	.	.	.	.	.	.	.	.
5	DPK762	.	.	.	.	.	.	.	.	.	.	.	.	.	.	.	.	.	.	.	.	.
4a	EF1237Blo1	.	.	.	.	.	.	.	.	.	.	.	.	.	.	.	.	.	.	.	.	G
4b	CAS08-0425	.	.	.	.	.	.	.	.	.	.	.	.	.	.	.	.	.	.	.	.	G
4b	DPK305	.	.	.	.	.	.	.	.	.	.	.	.	.	.	.	.	.	.	.	.	G
4b	DPL1021	.	.	.	.	.	.	.	.	.	.	.	.	.	.	.	.	.	.	.	.	G
1	B1012M	K	.	.	.	.	.	.	.	.	.	.	.	.	.	.	.	.	.	M	.	.
1	M7	.	.	.	.	.	.	.	.	.	.	.	.	.	.	.	.	.	.	N	.	.
6a	P35-2	.	.	.	.	I	.	.	.	.	.	.	.	.	.	E	.	.	.	.	.	.
6b	CAS08-0016	.	.	.	.	I	.	.	.	.	.	.	.	.	.	E	.	.	.	.	.	.
6b	DPL245	.	.	.	.	I	.	.	.	S	.	.	.	.	.	E	.	.	.	.	.	.
2a	CST35	.	.	.	.	.	L	.	I	S	.	.	.	V	.	E	N	.	.	N	.	.
2a	EB0911Sto	.	.	.	.	.	L	.	I	S	.	.	.	V	A	E	N	.	.	N	.	.
2b	SAT_BAL01	.	.	.	.	.	L	.	I	S	.	.	.	.	.	E	N	K	.	.	.	.
3	DSY562	.	S	.	.	.	.	.	.	.	.	.	N	.	.	E	N	.	S	.	.	.
3	M12	.	S	.	.	.	L	.	.	.	.	.	N	.	.	E	N	.	S	.	.	.
7	BG2	.	S	**N**	.	.	.	.	.	.	.	**I**	.	.	.	.	.	.	.	.	.	.
7	CST110	.	S	**N**	.	.	.	.	.	.	.	**I**	.	.	.	.	.	.	.	.	.	.
7	M6	.	S	**N**	.	.	.	.	.	.	.	**I**	.	.	.	.	.	.	.	.	.	.

Figure S6. Sequence analysis of the *SIR3* gene in all clades of *C. glabrata*. Non-synonymous mutations in each strain relative to the CBS138 reference genome are indicated by the amino acid listed. BG2 and other members of Clade-7 contain substitutions at codons 268 and 503 shown previously to be required for silencing of subtelomeric gene expression.
